# Hepatokines modulation in obesity: which exercise training model is better in men with obesity?

**DOI:** 10.3389/fendo.2025.1560453

**Published:** 2025-11-18

**Authors:** Ali Ataeinosrat, Hossein Abednatanzi, Shohreh Hajesfandiari, Maryam N. AL Nasser, Marjan Mansouri Dara, Parvin Farzanegi, Mahnoosh Salari Nahand, Abdullah Almaqhawi, Niloofar Karimi, Behnam Bagherzadeh-Rahmani, Ayoub Saeidi, Ismail Laher, Hassane Zouhal

**Affiliations:** 1 Department of Exercise Physiology, University of Tehran, Tehran, Iran; 2 Department of Physical Education and Sport Science, Science and Research Branch, Islamic Azad University, Tehran, Iran; 3 Department of Biological Sciences, College of Science, King Faisal University, Hofuf, Al-Ahsa, Saudi Arabia; 4 Department of Physical Education & Sports Sciences, Islamic Azad University, Sari, Iran; 5 Department of Education Sciences, Farhangian University, Tehran, Iran; 6 Department of Family and Community Medicine, College of Medicine, King Faisal University, Al Hofuf, Saudi Arabia; 7 Department of Exercise Physiology, Faculty of Sport Sciences, University of Mazandaran, Mazandaran, Babolsar, Iran; 8 Department of Exercise Physiology, Faculty of Sports Sciences, Shomal University, Amol, Iran; 9 Department of Physical Education and Sport Sciences, Faculty of Humanities and Social Sciences, University of Kurdistan, Sanandaj, Kurdistan, Iran; 10 Department of Anesthesiology, Pharmacology and Therapeutics, The University of British Columbia, Vancouver, BC, Canada; 11 International Institute of Sport Sciences (2I2S), Rennes, France

**Keywords:** exercise, Tabata, HIIT, obesity, hepatokines

## Abstract

**Objective:**

Obesity is associated with an exacerbated metabolic condition that is related to impaired secretion of hepatokines. This study aimed to compare the effects of 12 weeks continuous aerobic, Tabata, and high intensity interval training (HIIT) on the levels of fetuin-A, fetuin-B, fibroblast growth factor 21 (FGF-21), plasminogen activator inhibitor-1 (PAI-1), fibrinogen-like protein 1 (FGL1), and selenoprotein P.

**Methods:**

44 obese males were randomly divided into four groups (n=11): control group (CT), endurance training group (ET), Tabata group (Tabata), and HIIT group (HIIT). Exercise training programs lasted for 12 weeks/three sessions per week. Each session lasted 60 minutes, containing warm-up (10 minutes), main training for each group (40 minutes), and ending with cooling down (10 minutes). Measurements were made12 and 48 h before the initiation of the main interventional protocols, and then again 48 h after the final session of the training protocol.

**Results:**

Baseline data were similar in all groups (p>0.05). There was a significant group-by-time interaction for fetuin B (p=0.0004), FGF-21 (p=0.007), FGL-1 (p=0.0139), weight (p=0.0110), BMI (p=0.009), %fat (p<0.0001) and selenoprotein-1 (p=0.0052). There was a main effect of time for fetuin A and PAI-1 (p<0.0001). The reductions in fetuin B were greater in HIIT vs. CT (mean diff: -0.13) and Tabata vs. CG (mean diff: -0.14) (p<0.05), while decreases in FGF-21 were greater in ET vs. CT (mean diff: -19.1), HIIT vs. CT (mean diff: -26.22)(p<0.05), and Tabata vs. CT (mean diff: -22.8). Reductions in FGL-1 were greater in ET vs. CT (mean diff: -11.5)(p<0.05), HIIT vs. CT (mean diff: -8.1), and Tabata vs. CT (mean diff: -11.3)(p<0.05).

**Conclusion:**

Performing 12 weeks of physical activity led to decreases in weight, BMI, %fat, fetuin-B, FGF-21, FGL-1, and selenoprotein P, and greater reduction observed in the Tabata and the HIIT groups.

## 1 Introduction

The liver can respond to nutritional excess and deficiency status by regulating glucose and lipid metabolism through lipoprotein synthesis, glycogenolysis, and gluconeogenesis (1). The liver also releases hepatokines (liver secreted proteins) such asfetuin-A, fetuin-B,adropin, activinE, follistatin, and angiopoietin-like proteins (ANGPTLs),which can either worsen or improve metabolic conditions (2). Hepatokines can communicate with distant sites such as adipose tissue, skeletal muscle, and the central nervous system (CNS), and dysregulation of hepatokines can disrupt energy homeostasis and cause the metabolic dysfunction (3). In this context, changes in hepatokine levels occur in the obesity and diabetes (4).

Obesity results from positive energy balance (caloric consumption exceeds energy expenditure)over long periods, and is major risk factor for metabolic syndrome, type 2 diabetes mellitus (T2D), cardiovascular disease, atherosclerosis, dyslipidemia, hypertension, nonalcoholic fatty liver disease (NAFLD), and some types of cancers (5). Alterations in cytokines secreted from the liver (hepatokines), adipose tissues (adipokines) and skeletal muscles (myokines), act in an autocrine, paracrine or endocrine manner (6). Fetuin is a member of the cystatin super family protease inhibitors, firstly isolated from bovine serum, and consists of fetuin A and fetuin B, with important roles in various physiological and pathological process (7). Fetuin-A, also known as α-Heremans-Schmid glycoprotein (AHSG), is predominantly secreted by hepatocytes into the blood circulation (8). The human homolog of fetuin-A is a protein with 349 amino acids and molecular weight between 52–60 kDa (9). In addition to secretion by the liver, lesser amounts of fetuin A secretion are also released by other organs such as the tongue, placenta, and adipose tissues (10, 11). Higher circulating levels of fetuin-A can affect obesity, metabolic syndrome, and insulin resistance/T2D (12), and fetuin A has roles in the development of T2DM, metabolic disorders, NAFLD, cardiovascular diseases (CVD), some types of cancers, and some brain disorders (13).

Fetuin-B is the second member of the fetuin family in the mammals (14), with a 22% homology with fetuin A (10). Fetuin-Bis a adipokine/hepatokine that is mostly secreted by the liver and adipose tissue, and its levels increase with impaired insulin action and during glucose intolerance (15). As is the case with fetuin A, elevation levels of fetuin B also occur in diabetes and metabolic syndrome (16).

Fetuin-Ais a novel risk factor for endothelial dysfunction (17), and its levels correlate with changes in interleukin-6 (IL-6), IL-18, tumor necrosis factor α (TNFα), plasminogen activator inhibitor-1 (PAI-1), leptin, and resistin, all of which play are involved in the pathophysiology and inflammatory process of diabetic kidney disease (18). Fetuin A increases the expression of pro inflammatory cytokines including PAI-1 in perivascular fat cells (19). PAI-1, a member of the serine proteinase inhibitors (serpin) super family that is secreted by the liver (hepatokine), activates the coagulation system during athero-thrombosis, with important roles in CVDs (20, 21). In addition to the liver, PAI-1 production also occurs in adipocytes, cardiac myocytes, smooth muscle cells, endothelial cells, platelets, and monocytes/macrophages (22). Circulating levels of PAI-1 increase in obesity, T2D, atherosclerosis, thrombosis, and some types of cancer (23).

Fibrinogen-like protein 1 (FGL-1),also known as hepatocyte-derived fibrinogen-related protein-1 (HFREP-1) or hepassocin, has mitogenic effects on hepatocytes (24). Adipose tissue can also express FGL-1 as a regulator of lipid metabolism (25). Hepatic expression of FGL-1 increases in the high-fat diet–fed mice with NAFLD, in turn led to activation of ERK1/2 in order to facilitate lipogenesis, ultimately is associated with development of hepatic steatosis (26). Levels of FGL-1 are higher in overweight and obese subjects, and are positively correlated with BMI, waist circumference, degree of obesity, and insulin resistance, suggesting thatFGL-1 may link obesity, diabetes and NAFLD (27). In addition, levels of FGL-1 and body fat percentage are positively correlated, and suggesting that FGL-1 as therapeutic target in obesity (28). Collectively, there is much evidence that hepatokines are dysregulated in metabolic disorders and cardiovascular diseases (29).

Different types of exercise training are used to manage overweight and obesity in adults (30), but their effects can be different. Moderate-intensity continuous training (MICT) and high-intensity interval training (HIIT) produce similar improvements in weight loss and cardiovascular risk factors, while HIIT has additional benefits in cardiorespiratory fitness (31), and is considered a time-efficient weight management exercise (32). Tabata is one of the most popular forms of shorter HIIT workouts (33), and reduces body fat in healthy sedentary subjects (34). The effects of different types of exercise training, especially Tabata training, on the levels of hepatokines as a possible mechanism for mediating the benefits of exercise in overweight and obese subjects are unclear. We compared the effects of 12 weeks MICT, Tabata, and high intensity interval training (HIIT) on levels of fetuin-A, fetuin-B, FGF-21, PAI-1, FGL-1, and selenoprotein P in obese men. Therefore, this study was designed to show which exercise training model has the greatest effect on levels of fetuin-A, fetuin-B, FGF-21, PAI-1, FGL-1, and selenoprotein P in obese men.

## 2 Material and methods

### 2.1 Participants

**Figure 1:**
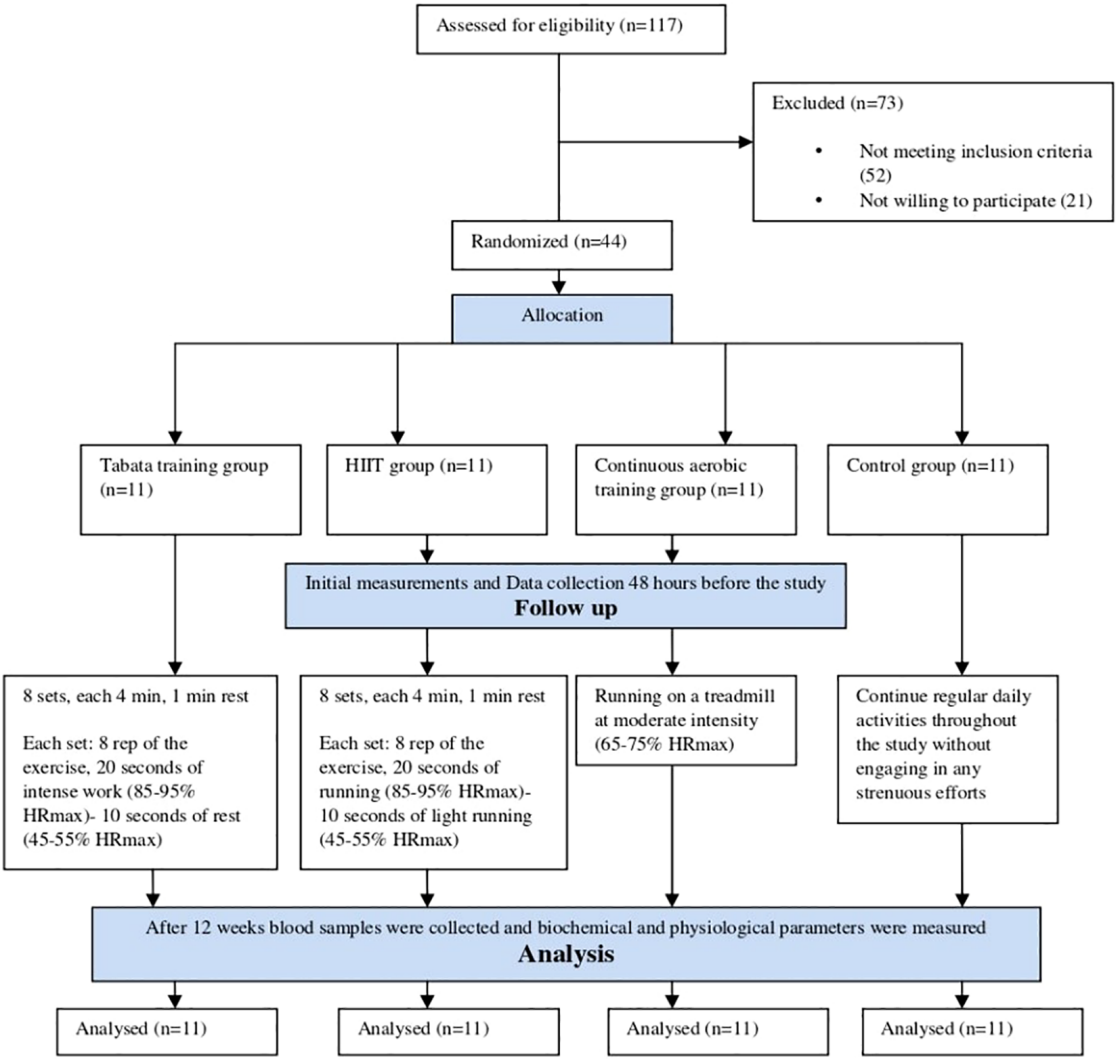
CONSORT flow diagram.

Subjects in this study were males aged 21 to 35 years with obesity. The decision to enroll only male participants was intentional to minimize biological variability related to sex-specific factors, including menstrual-cycle–related hormonal fluctuations, contraceptive use, menopausal status, and age-related comorbidities or medications that could confound hepatokine and metabolic responses. This approach aimed to improve internal validity for detecting exercise-induced changes, though we acknowledge that it limits generalizability to women and older adults. A total of 117 volunteers were recruited through social media and in-person outreach. Participants received documentation outlining the study’s aims and methods, and they were asked to complete health and physical activity questionnaires, as well as provide written consent. The inclusion criteria (I) Body mass index (BMI) > 30 kg/m²; (II) Waist-to-Height Ratio (WHtR) > 0.5; (III) non-consumption of alcohol and smoking; (IV) no regular intense physical activities or exercise during the last six months; (V) no chronic diseases; (VI) no consumption of any medications or supplements; and (VII) no somatic injuries. Subjects were excluded if they were unable to continue in the study, did not attend training sessions for two or three consecutive days, or if they were injured or fell ill during the study period. All study participants were examined by a cardiologist, resulting in a final sample size of 44 individuals with a mean age of 27.32± 3.20 years, height of 177.47 ± 2.97 cm, weight of 101.03± 3.09 kg, and BMI of 32.08 ± 1.04 kg/m². No participants were withdrawn from the study as a result of musculoskeletal injuries. Subjects were randomly divided into four groups of 11 participants each: a control group (n=11), Tabata training group (n=11), a HIIT group (n=11), and a continuous aerobic training group (n=11), by adaptive randomization method. Eligibility was assessed and 44 participants were randomized to four arms (n=11 per arm) using covariate-adaptive minimization with a random component to balance on age and BMI. Strata were defined as age (21–28 vs 29–35 years) and BMI (30–32.9 vs 33–38). The minimization procedure was executed by an independent statistician using R (version 4.1.3) to generate a computer-based sequence with a predefined imbalance-minimizing probability. Allocation concealment was ensured via centralized, web-based randomization; the enrolling investigators had no access to upcoming allocations. Study participants were free to leave the study whenever they wished (**Figure 1**). The study was approved by the National Research and Ethics Committee of Islamic Azad University (Ethics code: IR.IAU.AMOL.REC.1402.070).

Overall, 117 volunteers were assessed for eligibility. Of these, 44 met the predefined inclusion criteria and were randomized into four groups (n = 11 per group); the remaining 73 individuals were either ineligible (n = 28) or declined participation (n = 45). Reasons for ineligibility at screening (n = 28) were as follows: failure to meet anthropometric criteria (BMI/WHtR) (n = 10), recent regular structured exercise (n = 6), medical contraindications (cardiovascular/hepatic/renal disease or diabetes) (n = 4), abnormal screening laboratory results (n = 2), current use of excluded medications or supplements (n = 3), and logistical issues (n = 3). A CONSORT flow diagram summarizing recruitment, screening, allocation, follow−up, and analysis is provided in **Figure 1**. All participants underwent baseline medical and cardiology screening prior to enrollment. Exercise sessions were supervised by trained personnel and intensity was continuously monitored using Polar Team 2 chest-belt heart-rate monitors. Supervisors recorded attendance and any symptoms or adverse events at each session; these were entered into an adverse-event log and reviewed by the principal investigator. No serious adverse events or study withdrawals attributable to injury were observed.

### 2.2 Training protocols

**Figure 2:**
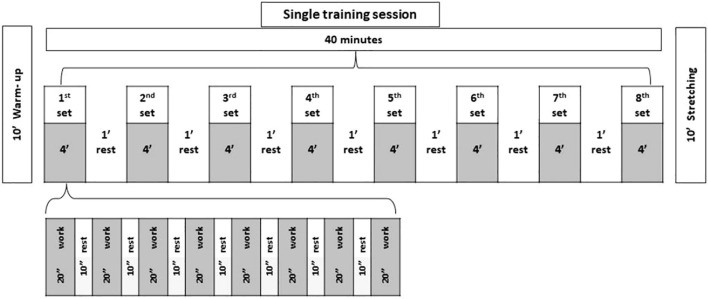
Scheme of the training sessions.

Training programs occurred over 12 weeks, with three sessions per week. Each session lasted 60 minutes, comprising a 10-minute warm-up, 40 minutes of main training for each group, and a 10-minute cool-down period (35) (**Figure 2**).

#### 2.2.1 Tabata training

Participants in this group completed eight training sets, each lasting 4 minutes, with 1 minute of rest between sets. Each set included eight repetitions of the exercise, featuring 20 seconds of intense work (85-95% HRmax) followed by 10 seconds of rest (45-55% HRmax) (35).

Each training set included the following exercises: 1) lower limb muscles (squats and jumps); 2) back muscles (back extensions); 3) rectus abdominal muscles (crunches); 4) chest muscles (push-ups); 5) arm muscles (triceps dips); 6) oblique abdominal muscles (side crunches); 7) shoulder girdle muscles (military press with a medicine ball); and 8) trapezius muscles (chin-ups) (36). The final 10 minutes of each session consisted of light stretching. The intensity of each session was monitored by measuring heart rates using Polar Team 2 monitors (Kempele, Finland).

#### 2.2.2 High-intensity interval training

Participants in this group completed eight training sets lasting 4 minutes, with 1 minute of rest between each set. Each set consisted of eight repetitions of running, with 20 seconds of intense work (85-95% HRmax) followed by 10 seconds of light running (45-55% HRmax).

#### 2.2.3 Continuous aerobic training

Exercise protocols consisted of running on a treadmill at moderate intensity (65-75% HRmax). The intensity of the training was monitored by changes in heart rate using Polar Team 2 monitors (Kempele, Finland).

#### 2.2.4 Control group

Participants in the control group were monitored for 12 weeks, during which they continued their normal daily activities without engaging in any strenuous efforts.

#### 2.2.5 Adherence and heart−rate monitoring

Participants completed a 12−week supervised program (3 sessions per week; 36 scheduled sessions per participant). Session attendance was recorded by the session facilitator and reasons for any absences were documented; the vast majority of participants attended the scheduled sessions with only sporadic absences, and no participant was withdrawn due to musculoskeletal injury. Per-group attendance was CT 97%, ET 95%, Tabata 99%, HIIT 96%.

Exercise intensity and training volume were prescribed relative to each participant’s maximal heart rate (HRmax). Heart rate was continuously recorded during all supervised sessions using Polar Team 2 chest-belt monitors (Polar Electro, Kempele, Finland). HRmax was estimated at baseline using the conventional formula (HRmax = 220 − age). Session HR data were expressed as %HRmax and used to ensure adherence to the intended intensity. The average session intensity across the three training groups was calculated by averaging %HRmax values recorded during each session across all participants within each group. Additionally, individual session data were analyzed to ensure compliance with the prescribed intensity range (65–75% HRmax) throughout the intervention period. All attendance logs and HR recordings were stored securely for further analysis.

Throughout the 12-week intervention, adverse events and symptoms were documented at each supervised session by trained facilitators. These logs were reviewed weekly by the principal investigator to ensure participant safety and adherence to the protocol. While serious adverse events were not observed during the study, the lack of independent adjudication of adverse events remains a limitation.

### 2.3 Anthropometry and body composition measurements

Body weight and height were measured using a calibrated scale (Seca, Germany) and a stadiometer (Seca, Germany), respectively. These measurements were then used to calculate body mass index (BMI, kg/m²).

### 2.4 Nutrient intake and dietary analysis

Three-day food records (two weekdays and one weekend day) were obtained before and after the study to assess changes in habitual dietary intake over time. Each food item was individually entered into Diet Analysis Plus version 10 (Cengage, Boston, MA, USA), allowing for the calculation of total energy consumption and the amount of energy derived from proteins, fats, and carbohydrates (**Table 1**).

**Table 1:** Mean ( ± SD) values of nutritional intake in the four study groups.

	Control		Continuous aerobic		HIIT		Tabata	
	Pre	Post	Pre	Post	Pre	Post	Pre	Post
Energy (kcal/d)	2249 ± 58	2258 ± 77	2269 ± 93	2198 ± 143	2261 ± 127	2199 ± 149	2267 ± 164	2195 ± 179
Carbohydrates (g/d)	279 ± 11.9	283 ± 18.7	281.4 ± 25.1	271 ± 36.7	279 ± 37.6	258 ± 26.2	290 ± 30.6	272 ± 21.1
Fats (g/d)	82.1 ± 9.7	83 ± 7.6	84.4 ± 10.8	76 ± 12.3	81.8 ± 13.9	73.2 ± 14.5	80.7 ± 11.83	71.6 ± 14.5
Proteins (g/d)	104 ± 10.1	106 ± 11.4	102 ± 13.6	95 ± 10.7	103 ± 14.7	94 ± 16.3	105 ± 13.9	88 ± 17.1

HIIT, High intensity interval training.

### 2.5 Blood analysis

Due to the nature of the supervised exercise interventions, participants and session facilitators were not blinded to group allocation. Outcome assessors performing anthropometry,body −composition measurements, and laboratory analyses were blinded to group assignment; biological samples and records were coded to preserve blinding. Measurement procedures were standardized and equipment calibrated, and assessors followed pre−specified protocols to minimize detection bias. In addition, several primary outcomes were objectively measured (e.g., continuously recorded heart rate), further reducing the potential for measurement bias. While participant blinding was not feasible, this limitation has been acknowledged as a potential source of bias, and steps were taken to mitigate its impact through rigorous standardization and objective outcome measurements. The first blood samples were collected 48 hours before the start of the exercise protocols and the second samples were collected 48 hours after 12 weeks of training programs. Blood samples were drawn from the right brachial vein and after an overnight fast. Samples were collected in tubes containing EDTA and centrifuged at 3000 rpm for 10 min to separate plasma which was stored at −70 °C until subsequent analysis. Blood samples were drawn between 8–10 am. Plasma markers were measured using ELISA kits having the following characteristics:

Fetuin A human elisakit(R&D Systems Quantikine, Minneapolis, Minnesota, USA). Catalogue No: #DFTA00. Intra-CV = 4.3%, inter-CV = 7.3%.
FetuinBhumanelisakit(Biovendor, Karasek, Czech Republic). Catalogue No: #RD191172200R. Intra-CV = 3.7%, inter-CV= 5.2%.
PAI-1 human elisakit(Trinity Biotech USA, St. Louis, MO). Catalogue No: # 210221. Intra-CV = 2.9%, inter-CV = 3.3%.
FGL1 human elisakit(Cusabio, Wuhan, China). Catalogue No: CSB-EL008653HU. Intra-CV <8%, inter-CV <10%.
FGF-21 human elisakit(Biovendor, Czech Republic). Catalogue No: RD191108200R. Intra-CV = 2.0%, inter-CV = 3.3%.
Selenoprotein P human ELISA kit (USCN Life Science, Wuhan, China) with an intraassay coefficient of variation of 6.7% and an interassay coefficient of variation of 4.7%.

### 2.6 Statistical analysis

The sample size justification was based on prior interval−exercise research using G*Power 3.1.9.2. Prior studies indicated a meaningful reduction in fetuin−A with an effect size (ES) of ~0.45. Using an F−test for repeated−measures ANOVA with within–between interaction (α = 0.05, power = 0.95), a minimum total sample of 28 participants (n = 7 per group) was required; to allow for potential disruptions related to COVID−19 and limited prior data on the studied hepatokines, the per−group sample was increased to 11 (N = 44) to preserve statistical power. All variables were inspected for normality using the Shapiro–Wilk test prior to inferential analyses. There were no missing values at any time point. Baseline characteristics are presented as mean ± SD. For longitudinal comparisons, a two × four repeated−measures ANOVA (time: pre vs. post × group: CT vs. ET vs. HIIT vs. Tabata) was used to evaluate main effects and time × group interactions. Sphericity was assessed with Mauchly’s test and, when violated, Greenhouse–Geisser corrections were applied to the degrees of freedom. When a significant time × group interaction was detected, between−group post−hoc comparisons were conducted using Sidak correction for multiple testing. Effect sizes are reported consistently throughout the manuscript: partial eta−squared (ηp2) is presented for omnibus ANOVA main effects and interactions, and standardized mean differences are reported for pairwise/post−hoc comparisons as Cohen’s d (with Hedges’ g provided where small−sample bias may affect estimates). Ninety−five percent confidence intervals (95% CI) for pairwise effect sizes and mean differences are provided in the Results and Tables. Conventional interpretation thresholds are stated in Methods (ηp2: small ≈0.01, medium ≈0.06, large ≥0.14; Cohen’s d: small ≈0.2, medium ≈0.5, large ≥0.8). All tests were two−tailed and a significance threshold of p < 0.05 was used. Analyses and effect−size calculations were performed using GraphPad Prism v8.4.3 and verified with supplementary computations.

## 3 Results

### 3.1 Participant characteristics

All variables met the assumptions for parametric testing: distributions were approximately normal (Shapiro–Wilk test, p > 0.05 for all variables) and variances were homogeneous across groups (Levene’s test, p > 0.05). One hundred and twelve participants were assessed for eligibility. Twenty-eight did not meet the inclusion criteria, while 36 were not interested in participating after the first interview. One participant from each group withdrew from the study for reasons of a lack of time, not interestedin the study, COVID-19, or musculoskeletal injury. There were no significant between-group differences in all baseline characteristics. The characteristics of the subjects in the experimental and control groups are shown in (**Table 2**). Participants’ age, height, weight, BMI and body fat percent did not differ at baseline.

**Table 2:** Characteristics of experimental and control subjects in men with obesity.

Groups	Age (years)	Height (Cm)	Weight (Kg)	BMI (Kg/m2)	BFP (%)
Continuous aerobic	27.5 ± 6.2	178.25 ± 3.18	101.55 ± 4.15	31.95 ± 1.00	35.24 ± 3.19
Tabata	26.4 ± 5.4	176.62 ± 2.58	100.92 ± 2.58	32.37 ± 1.26	35.20 ± 2.34
High intensity interval	28.1 ± 4.8	176.36 ± 3.18	100.91 ± 2.53	32.46 ± 1.17	34.37 ± 2.78
Control	27.3 ± 5.1	178.67 ± 2.60	100.73 ± 3.23	31.54 ± 0.41	34.10 ± 2.78
P-Value	0.897	0.173	0.936	0.149	0.705

BFP, body fat percent; BMI, body mass index.

#### 3.1.3 Body compassion and dietary monitoring

Analysis of the 3-day dietary records showed no significant between-group or pre-to-post changes in total energy intake or macronutrient composition during the intervention (all p>0.05) (**Table 1**).

As indicated in **Table 3**, there was a significant group × time interaction on weight [F = 4.22, p = 0.011], with significant decreases observed in the Tabata and high-intensity interval groups. Additionally, significant changes were noted in the exercise group, which exhibited a significant effect of time [F = 16.44, p = 0.001] and a significant effect of group [F = 4.05, p = 0.013]. The weight-related effect size for between-group effects was large (partial ηp2 = 0.233). The weight variable in the training groups showed a significant decrease compared to the control group (p = 0.017, 95% CI; 0.362 to 5.237, and p = 0.043, 95% CI; 0.054 to 4.92 for weight in the Tabata and high-intensity interval training groups, respectively). Furthermore, there was no significant difference between the continuous aerobic and control groups (p = 0.338, 95% CI; 0.776 to 4.10).

**Table 3:** Changes in body composition and the levels of biochemical markers in the research groups.

Stages								
Variables and groups	Pre-test M ± SD ^a^	12^th^ week M ± SD ^a^	Percentage of changes	P-value^b^	Time p-value	Group p-value	Time × group p-value	Partial eta squared
Weight (kg)								
Continuous aerobic	101.55 ± 4.15	97.95 ± 2.45	-3.67	0.052	0.001	0.013	0.011	0.233
High intensity interval	100.91 ± 2.53	96.93 ± 3.44	-4.10	0.006				
Tabata	100.92 ± 2.58	96.30 ± 2.31	-4.79	0.001				
Control	100.73 ± 3.23	102.09 ± 3.06	1.33	0.403				
BMI (kg/m2)								
Continuous aerobic	31.95 ± 1.00	30.85 ± 1.36	-3.56	0.054	0.001	0.784	0.009	0.026
High intensity interval	32.46 ± 1.17	31.20 ± 1.91	-4.03	0.006				
Tabata	32.37 ± 1.26	30.88 ± 1.06	-4.82	0.001				
Control	31.54 ± 0.41	32.00 ± 1.45	1.43	0.374				
BFP (%)								
Continuous aerobic	35.24 ± 3.19	32.96 ± 3.02	-6.91	0.036	0.001	0.005	0.001	0.272
High intensity interval	34.37 ± 2.78	31.29 ± 2.34	-9.84	0.018				
Tabata	35.20 ± 2.34	29.29 ± 1.81	-20.17	0.001				
Control	34.10 ± 2.78	36.76 ± 3.33	7.23	0.025				
Fetuin A(pg/ml)								
Continuous aerobic	4.79 ± 1.56	4.24 ± 1.67	-12.97	0.518	0.025	0.291	0.072	0.158
High intensity interval	5.40 ± 1.11	3.95 ± 1.62	-36.70	0.02				
Tabata	5.29 ± 1.45	3.57 ± 1.28	-48.17	0.014				
Control	4.89 ± 1.82	5.51 ± 1.29	11.25	0.385				
Fetuin B (pg/ml)								
Continuous aerobic	0.46 ± 0.14	0.37 ± 0.12	-24.32	0.169	0.001	0.014	0.001	0.358
High intensity interval	0.47 ± 0.14	0.30 ± 0.12	-56.66	0.007				
Tabata	0.46 ± 0.12	0.28 ± 0.15	-64.28	0.001				
Control	0.47 ± 0.12	0.57 ± 0.13	17.54	0.04				
FGL1(ng/ml)								
Continuous aerobic	47.11 ± 10.22	36.34 ± 7.54	-29.63	0.013	0.001	0.001	0.014	0.231
High intensity interval	51.17 ± 9.30	39.13 ± 7.20	-30.76	0.02				
Tabata	50.71 ± 11.31	33.12 ± 12.77	-53.10	0.008				
Control	51.06 ± 8.09	55.48 ± 10.81	7.96	0.426				
FGF21 (pg/ml)								
Continuous aerobic	193.54 ± 18.18	185.47 ± 12.92	-4.35	0.274	0.006	0.001	0.007	0.259
High intensity interval	195.09 ± 23.78	169.76 ± 14.23	-14.92	0.018				
Tabata	203.77 ± 21.16	167.80 ± 15.24	-21.43	0.004				
Control	202.36 ± 31.29	214.94 ± 18.90	5.85	0.353				
Sel1 (ng/ml)								
Continuous aerobic	798.94 ± 126.51	711.31 ± 103.06	-12.31	0.094	0.001	0.001	0.005	0.270
High intensity interval	778.67 ± 85.17	643.32 ± 73.78	-21.03	0.007				
Tabata	797.33 ± 109.26	601.81 ± 101.96	-32.48	0.001				
Control	816.20 ± 131.98	846.30 ± 60.05	3.55	0.549				
PAI (ng/ml)								
Continuous aerobic	26.75 ± 7.77	23.05 ± 4.42	-16.05	0.226	0.006	0.096	0.067	0.162
High intensity interval	25.46 ± 10.29	19.29 ± 4.45	-31.98	0.116				
Tabata	26.64 ± 9.95	17.96 ± 4.76	-48.32	0.011				
Control	26.28 ± 7.28	28.34 ± 4.65	7.26	0.327				

a Values are expressed as mean ± standard deviation.

b Within-group P-value.

c Significant at P < 0.05.

Values are presented as mean ± standard deviation. BFP, Body fat percent; BMI, Body mass index; Sell, selenium; FGF-21, fibroblast growth factor 21; PAI-1, plasminogen activator inhibitor-1; FGL-1, fibrinogen-like protein 1 FGL1; CT, Control group; HIIT, High intensity interval training group; ET, Endurance training group.

There was a significant group × time interaction on BMI [F = 4.38, p = p = 0.009], with notable decreases observed in both the Tabata and high-intensity interval groups. Additionally, significant changes were seen in the exercise group, showing a significant effect of time [F = 16.18, p = 0.001], but not a significant effect of group [F = 0.357, p = 0.784]. The BMI-related effect size for between-group effects was small (partial ηp2 = 0.026). As a result, there were no statistically significant differences between the continuous aerobic, Tabata, high-intensity interval, and control groups (p = 0.962, 95% CI −0.884 to 1.621; p = 1.00, 95% CI -1.10 to 1.39; and p = 1.00, 95% CI -1.31 to 1.19, respectively; **Table 3**).

Our results revealed a statistically significant difference in the group × time interaction for body fat percentage [F = 14.15, p = 0.001]. Significant decreases were observed in the continuous aerobic, Tabata, and high-intensity interval groups, with a significant effect of time [F = 20.65, p = 0.001] and a significant effect of group [F = 4.97, p = 0.005]. Additionally, the effect sizes for between-group comparisons related to body fat percentage were large (partial ηp2 = 0.272). The results of the *post hoc* test indicated that body fat percentage in the Tabata and high-intensity interval groups was significantly reduced compared to the control group (p = 0.006, 95% CI: 0.70 to 5.67 and p = 0.036, 95% CI:0.112 to 5.08, respectively; **Table 3**). Furthermore, there was no significant difference between the continuous aerobic and control groups (p = 0.613, 95% CI: -1.155 to 3.817).

### 3.2 Biochemical markers

There was not a significant group × time interaction on Fetuin A[F = 2.50, p = 0.072]. Additionally, significant changes were seen in the exercise group, showing a significant effect of time [F = 5.40, p = 0.025], but not a significant effect of group [F = 1.28, p = 0.291]. The Fetuin A -related effect size for group × time interaction effects were large (partial ηp2 = 0.158). As a result, there were no statistically significant differences between the continuous aerobic, HIIT, Tabata compared to the control group (p = 0.717, 95% CI −0.51 to 1.88; p = 1.00, 95% CI -0.66 to 1.72; and p = 0.486, 95% CI -0.424 to 1.96, respectively).

Our results revealed a statistically significant difference in the group × time interaction for Fetuin B [F = 7.44, p = 0.001]. Significant decreased were observed in the Tabata and HIIT groups, with a significant effect of time [F = 11.83, p = 0.001] and a significant effect of group [F = 4.00, p = 0.014]. Additionally, the effect sizes for group × time interaction were large (partial ηp2 = 0.358). The results of the *post hoc* test indicated that Fetuin B in the Tabata and HIIT groups was significantly decrease compared to the control group (p = 0.042, 95% CI: 0.003 to 0.266 and p = 0.020, 95% CI: 0.016 to 0.279, respectively). Furthermore, there was no significant difference between the continuous aerobic and control groups (p = 0.188, 95% CI: -0.026 to 0.237).

Our results revealed a statistically significant difference in the group × time interaction for FGL1 [F = 4.00, p = 0.014]. Significant decreased were observed in the continuous aerobic, Tabata and HIIT groups, with a significant effect of time [F = 14.59, p = 0.001] and a significant effect of group [F = 8.97, p = 0.001]. Additionally, the effect sizes for group × time interaction were large (partial ηp2 = 0.231). The results of the *post hoc* test indicated that FGL1 in the continuous aerobic, HIIT and Tabata groups was significantly decrease compared to the control group (p = 0.001, 95% CI: 4.46 to 18.62; p = 0.017, 95% CI: 1.04 to 15.20 and p = 0.001, 95% CI: 4.27 to 18.43, respectively).

Our results revealed a statistically significant difference in the group × time interaction for FGF21 [F = 4.65, p = 0.007]. Significant decreased were observed in the Tabata and HIIT groups, with a significant effect of time [F = 8.32, p = 0.006] and a significant effect of group [F = 10.51, p = 0.001]. Additionally, the effect sizes for group × time interaction were large (partial ηp2 = 0.259). The results of the *post hoc* test indicated that FGF21 in the continuous aerobic, HIIT and Tabata groups was significantly decrease compared to the control group (p = 0.003, 95% CI: 4.94 to 33.34; p = 0.001, 95% CI: 12.02 to 40.42 and p = 0.001, 95% CI: 8.65 to 37.05, respectively).

Our results revealed a statistically significant difference in the group × time interaction for Sel1[F = 4.93, p = 0.005]. Significant decreased were observed in the Tabata and HIIT groups, with a significant effect of time [F = 20.35, p = 0.001] and a significant effect of group [F = 7.44, p = 0.001]. Additionally, the effect sizes for group × time interaction were large (partial ηp2 = 0.270). The results of the *post hoc* test indicated that Sel1in the HIIT and Tabata groups was significantly decrease compared to the control group (p = 0.002, 95% CI: 34.38 to 206.12 and p = 0.001, 95% CI: 45.80 to 217.54, respectively). Furthermore, there was no significant difference between the continuous aerobic and control groups (p = 0.110, 95% CI: -9.74 to 161.99).

There was not a significant group × time interaction on PAI [F = 2.57, p = 0.067]. Additionally, significant changes were seen in the exercise group, showing a significant effect of time [F = 8.28, p = 0.006], but not a significant effect of group [F = 2.26, p = 0.096]. The PAI-related effect size for group × time interaction effects were large (partial ηp2 = 0.162). As a result, there were no statistically significant differences between the continuous aerobic, HIIT, Tabata, and control groups (p = 1.00, 95% CI −3.82 to 8.64; p = 0.203, 95% CI -1.29 to 11.17 and p = 0.188, 95% CI -1.22 to 11.24, respectively).

## 4 Discussion

Our study indicates that 12-weeks of different types of exercise training (including HIIT, MICT, Tabata) decreased levels of cardiometabolic risk factors and circulating levels of fetuin-A, fetuin-B, FGF-21, PAI-1, FGL-1, and selenoprotein P in obese men. There were additional improvements in hepatokine levels in the HIIT and Tabata training groups compared to the MICT group. The liver is a central regulator of whole-body energy homeostasis, with hepatokines considered as an promising targets for the treatment of development of metabolic disorders (37). Exercise training modulates the levels of different organokines, including adipokines, myokines and hepatokines (38) and exercise induced changes in hepatokine levels have been suggested to ameliorate metabolic disorders such as obesity or T2D (39). We confirmed that the benefits of various type of exercise training are mediated by changes in hepatokine levels.

### 4.1 Fetuins

Our findings indicate reductions in fetuin A and fetuin B levels with HIIT, MICT and Tabata training. There are limited reported on the effects of exercise on fetuin levels (particularly fetuin B), but down regulation of both fetuin A and fetuin B with aerobic and resistance training have been reported (40). Studies report that exercise decreases (41), increases (42) and or causes no changes (43) of fetuin A levels. Fetuin A is a chemo-attractant secretory protein that stimulates the macrophage secretion by the liver and adipose tissue, and activated macrophage in turn cause increase the expression of pro-inflammatory cytokines (such as TNF-α and IL-6) which can impair glucose metabolism and result in obesity related disorders (44). Moreover, fetuin A is an important risk factor for insulin resistance related to Akt and phosphatidylinositide 3-kinase (PI3K) signaling pathways (19). In addition, fetuinA enhances insulin resistance by decreasing the expression of glucose transporter-4 (GLUT-4) proteins by activating toll-like receptor 4 (TLR-4) in skeletal muscles (45). Glucose and lipid metabolism is also affected by fetuin B, and significantly higher circulating levels of fetuin B occur inT2D patients (46). Furthermore, fetuin B induces a pro inflammatory response in adipocytes, and could mediate peripheral insulin resistance (45), which could lead to NAFLD (47). Activation of AMP-activated protein kinase (AMPK), a central energy sensor upregulated during exercise, enhances insulin receptor sensitivity and fatty acid oxidation, contributing to the downregulation of fetuin-A and selenoprotein P—both associated with impaired insulin signaling (48).

Targeting fetuinsis of interest because of their many adverse effects, as shown by enhanced insulin sensitivity, resistance to a high-fat diet and aging induced weight gain infetuin-A-knockout mice (49, 50). Our study demonstrates that exercise reduces fetuin A and fetuin B levels. Possible mechanisms for the lowering of fetuin A levels by exercise include: decreases in intrahepatic fat content by reduction of sterol regulatory element-binding protein (SREBP)-1cand enhanced peroxisome proliferator-activated receptor γ (PPAR-γ) expression; decreased hepatic glucolipotoxicity by reactive oxygen species (ROS); inhibition of pro-inflammatory mediators; and activation of Akt and Akt substrate of 160 kDa (AS160) phosphorylation, which in turn reduce insulin resistance (51). Exercise attenuates hepatic expression of fetuin A by high levels of free fatty acids (FFAs) by stimulation of NF-κB signaling (56), and reduces hyperglycemia increases hepatic expression by activating the ERK-1 and ERK-2 (61). Our results demonstrate that exercise reduces both glucose and fetuin A levels. Levels of fetuin-B levels in patients with metabolic syndrome are by improvements in insulin sensitivity, with a positive correlation between fetuin B and oxidative stress (16). Therefore, fetuin B reduction by exercise can be partly mediated by exercise induced reductions of oxidative stress (52).

### 4.2 Plasminogen activator inhibitor-1

PAI-1 inhibits PA (tissue-type plasminogen activator) and uPA (urinary-type plasminogen activator), and is a key regulator of fibrinolysis and cell migration (53). PAI-1 has endocrine functions, including in obesity. A positive energy balance increases the expression of PAI-1 in adipocytes, where it increases differentiation of adipocytes, causes adipocyte hypertrophy, and obesity. PAI-1 stimulates inflammatory pathways (including increasing the production of TNF-α) and decreases the expression of PPAR-γ, and promoting insulin resistance and obesity (23, 54).

Our findings indicate that different exercise training modalities can decrease PAI-1 levels. Several mechanisms have been proposed for the reductions in PAI-1 by exercise, including by increased endothelial integrity of vascular endothelium, and stimulating the release on of nitric oxide (NO),which suppresses PAI-1 production by vascular smooth muscle cells and platelets (55). Also, Exercise stimulates PPARs, particularly PPAR-α and PPAR-γ, which regulate genes involved in lipid metabolism and hepatic fat clearance, leading to suppressed expression of lipotoxic hepatokines such as fetuin-B and PAI-1 (54).

Our findings agree with those of Ahmad et al (56) who reported that eight weeks HIIT and MICT reduced PAI-1 levels women with T2D women, but with no difference between the HIIT and MICT groups likely because exercise of adaptation of skeletal muscles to the effects of PAI, including the gene expression and post-translational modifications (56). Exercise increases tPA protein levels and decreases PAI-1 levels in trained skeletal muscles, and thus increases local fibrinolytic status and blood flow to active muscles (57). However, in contrast to our findings, Ahmad et al. (58) reported that HIIT did not reduce PAI-1 levels more than the effects of MICT, probably due to the shorter duration of exercise (eight weeks) used by Ahmad et al. (58). A study by Bodary et al. (58) reported no changes in PAI-1 levels in overweight and obese subjects after 10 weeks moderate aerobic training (walking with 65 percent of reserve heart rate), and concluded that reductions in PAI-1 require decreases in adipose tissue and changes in metabolic variables (58). Our study indicate PAI-1 reductions simultaneous with decreases in adipose tissue, suggesting that exercise reduces PAI-1 levels by loss of adipose tissue (59, 60).

### 4.3 Fibrinogen-like protein 1

Our study also indicates that exercise reduced FGL-1 levels. FGL-1 is a novel hepatokine mainly expressed in the liver under normal physiological conditions, but whose levels are increased by high fat conditions and lead to lipid accumulation and inflammation, which in turn trigger the development of NAFLD, diabetes, and obesity (61). ERK1/2 is activated by FGL-1 to induce lipogenesis, and increased ERK1/2 phosphorylation stimulates C/EBPβ.FGL1 facilitates adipogenesisbyaERK1/2-C/EBPβ- dependent pathway, and targeting FGL-1 could be a novel therapeutic strategy in obesity and its related disorders (28). For example, FGL-1 plays an important role in insulin resistance and T2D by ERK1/2-dependentmechanisms, andFGL-1 up regulation could increase ERK1/2 activity, inhibit insulin signaling and lead to insulin resistance (62). There is a positive correlation between FGL-1 and obesity markers (31), and FGL-1 levels are reduced by laparoscopic sleeve gastrectomy (LSG) for weight loss (63), supporting our findings that exercise reduces decrease in the FGL-1 levels and also decreases body weight and adiposity. Decreases in FGL-1 levels by exercise training maybe intensity dependent, as demonstrated by Jokar et al. (64) where moderate and high intensity circuit resistance training, but not low intensity training, decreased FGL-1 levels likely related to loss of adipose tissue by exercise training (64). Although we observed adipose tissue loss after exercise training, we did not measure the FGL-1 expression in the adipose tissue.

### 4.4 Fibroblast growth factor 21

The adipo-hepatokine FGF-21 is a major energy regulator that affects ROS levels, endoplasmic reticulum stress and other cellular processes, and has favorable effects on body weight control and triglyceride or cholesterol levels (65). Despite the positive effects of FGF-21 on metabolism, up-regulation of FGF-21 and its correlation with BMI has been reported in obesity, leading to the suggestion FGF-21 resistance condition may occur in obesity (66). Since FGF-21 levels are correlated with adipose tissue mass, plasma glucose and insulin levels (insulin resistance) (67). However, exercise training may not always change FGF-21 levels, as exercise can sensitize FGF-21 actions without its concentrations (68). Although FGF−21 has beneficial actions, levels are paradoxically elevated in obesity and metabolic disease, often reflecting compensatory up−regulation and “FGF−21 resistance” (66). Therefore, reductions in plasma FGF−21 after chronic exercise may indicate improved metabolic health with reduced compensatory secretion (and improved FGF−21 sensitivity), rather than loss of benefit (67). Alternatively, lower FGF−21 could reflect reduced production independent of sensitivity (67). In our study FGF−21 declined alongside reductions in adiposity and cardiometabolic markers, favoring reduced compensation; however, without receptor/signaling, insulin−sensitivity or hepatic−fat measures, causal inference is limited.

### 4.5 Selenoprotein P

Selenoprotein P (SeP) is another protein secreted by the liver (69), and is transported to peripheral tissues (70). Levels of SeP are increased in overweight and obese people, indicating SeP secretion from adipose tissue (32). Therefore, decrease in adipose tissue by exercise may lower SeP release as shown in our study. SeP causes insulin resistance in hepatocytes and myocytes by several mechanisms, including suppressing auto-phosphorylation of insulin receptor, AKT pathways responding to insulin, decreasing insulin-induced cellular glucose uptake, suppressing the hepatic AMPK signaling pathways, and by disrupting insulin production by pancreatic beta cells (71, 72). Insulin resistance and liver function are closely linked to the physiological role of SeP, a hepatokine primarily synthesized in the liver (24, 26). SeP functions as a selenium transport protein, but it also plays a critical role in metabolic regulation. Elevated levels of SeP have been associated with impaired insulin signaling and glucose homeostasis, contributing to systemic insulin resistance (70, 71). In insulin-resistant states, such as type 2 diabetes and NAFLD, hepatic overproduction of SeP exacerbates metabolic dysfunction by inhibiting insulin action in peripheral tissues (24, 73). Furthermore, insulin normally suppresses SeP expression; however, in insulin-resistant conditions, this regulatory mechanism is impaired, creating a feedback loop that promotes further metabolic derangement (26, 32). Therefore, understanding the interplay between SeP, insulin resistance, and liver function provides key insights into the pathophysiology of metabolic disorders and highlights SeP as a potential biomarker and therapeutic target (72, 73).

Tabata training and short-term HIIT formats are popular forms of interval training, and can be used to increase aerobic and anaerobic fitness, reduce fat and even improve blood pressure, insulin sensitivity and glucose regulation in a relatively short time (33). Furthermore, even short term (four weeks) Tabata training reduces body fat percentage in obese subjects (74). Our findings indicate that HIIT and Tabata training appeared more effective than MICT in improving glycemic control and hepatokine profiles in obese adults.

## 5 Conclusion

Performing 12 weeks of Tabata and HIIT training decreased weight, BMI, fat percentage, fetuin-B, FGF-21, FGL-1, and selenoprotein P, levels, and it seems the benefits of Tabata and HIIT were more than MICT. The lack of significant changes in the control group over time can be attributed to their sedentary behavior and absence of metabolic stimulation. These hepatokines are sensitive to physiological stressors such as exercise, which modulate their expression through improvements in insulin sensitivity, inflammation, and liver metabolism. In contrast, the training group showed alterations in several of these markers, highlighting the role of physical activity in regulating hepatokine secretion.

## 6 Limitations

This study is subject to several methodological limitations. First, we used a predictive equation (HRmax = 220 − age) to estimate HRmax; this approach may over− or under−estimate true HRmax and thus introduce error into %HRmax calculations and exercise intensity classification. The relatively small sample size may have reduced statistical power and limits external validity. To minimize biological variability related to sex− and age−specific factors, we intentionally enrolled only male participants within a narrow age range (21–35 years): this choice aimed to reduce variability from menstrual−cycle–related hormonal fluctuations, contraceptive use, menopausal status, and age−related comorbidities or medications that could confound hepatokine and metabolic responses. While restricting the sample improved internal validity for detecting exercise−induced changes, it limits generalizability to women and older adults. Dietary intake was not standardized during the 12−week intervention, although three-day food records (two weekdays and one weekend day) were collected at baseline and post-intervention to assess changes in habitual dietary intake over time. These records were analyzed using Diet Analysis Plus version 10 (Cengage, Boston, MA, USA) to calculate total energy consumption and macronutrient distribution (proteins, fats, and carbohydrates). However, self-reported short-term records may not fully capture habitual intake, and residual confounding from uncontrolled dietary changes cannot be excluded. Glucose and insulin concentrations were not measured; therefore, references to glucose–insulin signaling as a potential mediator are hypothetical and were discussed only to provide mechanistic context based on prior literature. Future studies should include women (with appropriate cycle−phase or contraceptive stratification), older adults, more comprehensive metabolic and molecular profiling, controlled dietary conditions or repeated dietary monitoring, and formal safety surveillance to evaluate sex− and age−specific responses and underlying mechanisms. An important limitation is the lack of mechanistic biomarker assessment. Although we discuss pathways such as insulin signaling, AMPK activation, GLUT-4 translocation and oxidative stress, these mechanisms were not measured here and therefore remain speculative. Future studies should include direct measures of glucose homeostasis and insulin sensitivity (e.g., fasting glucose/insulin, HOMA-IR, OGTT or hyperinsulinemic-euglycemic clamp), hepatic fat quantification (e.g., MRI-PDFF), molecular markers of insulin/AMPK signaling and GLUT-4 (muscle or peripheral cells), and oxidative-stress/inflammatory biomarkers, with sampling at baseline, acute post-exercise and post-intervention time points to better define causal pathways. While adverse events were regularly documented and reviewed by the principal investigator, the lack of independent adjudication for safety monitoring represents a methodological limitation. Future studies should consider implementing independent safety committees to enhance the rigor of adverse event reporting and adjudication.

## Data Availability

The raw data supporting the conclusions of this article will be made available by the authors, without undue reservation.
